# Alzheimer-Demenz bei Menschen mit einem Down-Syndrom: Ergebnisse leitfadengestützter Expert:inneninterviews zu Versorgungsdefiziten in Diagnostik und Therapie sowie Lösungsansätzen

**DOI:** 10.1007/s00391-024-02371-8

**Published:** 2024-10-09

**Authors:** Theresa Hüer, Milena Weitzel, Godwin Denk Giebel, Pascal Raszke, Jürgen Wasem, Johannes Levin, Georg Nübling, Olivia Wagemann, Elisabeth Wlasich, Johannes Pantel, Valentina Tesky, Arthur Schall, Anke Walendzik

**Affiliations:** 1 Lehrstuhl für Medizinmanagement, Universität Duisburg-Essen, Thea-Leymann-Str. 9, 45127 Essen, Deutschland; 2 Neurologische Klinik und Poliklinik, Ludwig-Maximilians-Universität München, München, Deutschland; 3 Deutsches Zentrum für Neurodegenerative Erkrankungen (DZNE), Standort München, München, Deutschland; 4 Munich Cluster for Systems Neurology (SyNergy), München, Deutschland; 5 Institut für Allgemeinmedizin, Goethe-Universität Frankfurt am Main, Frankfurt am Main, Deutschland; 6 Essener Forschungsinstitut für Medizinmanagement GmbH, EsFoMed, Essen, Deutschland

**Keywords:** Interviewstudie, Medizinische Versorgung, Trisomie 21, Versorgungsdefizite, Lösungsansätze, Interview study, Medical care, Trisomy 21, Medical care deficits, Solution approaches

## Abstract

**Hintergrund:**

Menschen mit einem Down-Syndrom (MmDS) haben ein genetisch bedingt stark erhöhtes Risiko, an einer früh beginnenden Alzheimer-Demenz zu erkranken. In einer Interviewstudie mit Ärzt:innen, Patientenvertretungen sowie Mitarbeitenden in Wohn- und Arbeitseinrichtungen wurden Defizite im medizinischen Versorgungsprozess und Lösungsansätze erhoben.

**Methodik:**

Es wurden 14 teilstrukturierte Interviews durchgeführt und über eine qualitative Inhaltsanalyse ausgewertet.

**Ergebnisse:**

Fehlende Kenntnisse und Erfahrungen von Ärzt:innen im Umgang mit sowie in der medizinischen Versorgung von MmDS zeigten sich als zentrale Herausforderung. Zudem ist die Diagnostik der Demenz bei MmDS aufgrund verschiedener Ursachen (u. a. Fehlen geeigneter Diagnostikinstrumente in der Regelversorgung, fehlende zeitliche/finanzielle Ressourcen) erschwert. Zweifel an der Wirksamkeit von Antidementiva wurden geäußert sowie Hintergründe für einen erhöhten Einsatz sedierender Medikamente diskutiert.

Eine aufmerksame Verhaltensbeobachtung und eine Einbindung von Betreuenden, eine regelmäßige Überprüfung und Reduktion von Multimedikation sowie der Einsatz alternativer Verhaltensmodifikationstechniken wurden als Lösungsansätze genannt.

**Schlussfolgerung:**

Die identifizierten Defizite in der medizinischen Versorgung der Zielpopulation sowie die Lösungsansätze gehen in die Entwicklung gesundheitspolitischer Handlungsempfehlungen ein, um die Versorgungssituation nachhaltig zu optimieren.

## Abstract (en)

**Background:**

People with Down syndrome have a genetically increased risk of developing early onset Alzheimer’s dementia. An interview study with healthcare providers, patient representatives and employees in residential and work facilities was conducted to identify deficits in the healthcare process and approaches to overcoming them.

**Method:**

In this study 14 semi-structured interviews were conducted and analyzed using qualitative content analysis.

**Results:**

A lack of knowledge and experience on the part of medical service providers in dealing with and providing medical care for people with Down syndrome was identified as a key challenge. In addition, the diagnosis of dementia in people with Down syndrome is difficult for various reasons (including lack of appropriate diagnostic tools in standard care and lack of time or financial resources). Doubts were expressed about the efficacy of antidementia medications and the reasons for the increased use of sedatives were discussed. Attentive observation of behavior and involvement of caregivers, regular review and reduction of polypharmacy and the use of alternative behavior modification techniques were mentioned as possible solutions.

**Conclusion:**

The identified deficits in the medical care of the target population and the approaches to solving them will be incorporated into the development of health policy recommendations in order to optimize the care situation of those affected in the long term.

Menschen mit einer Trisomie 21 (auch Down-Syndrom (DS) genannt) haben ein stark erhöhtes genetisches Risiko, an einer früh beginnenden Alzheimer-Demenz zu erkranken. Grund dafür ist ein für die Alzheimer-Krankheit verantwortliches Gen auf dem Chromosom 21, welches für das Amyloidvorläuferprotein (APP) codiert und bei den meisten Menschen mit DS (MmDS) 3fach vorhanden ist. APP ist das Vorläufereiweiß, aus dem die Bausteine der für die Alzheimer-Krankheit typischen Amyloidplaques ausgeschnitten werden. Aus der Triplikation und der damit einhergehenden relativen Überexpression dieses Gens resultiert die Akkumulation von Amyloidplaques im Gehirn [1]. Über 80 % der MmDS entwickeln voraussichtlich bis zum Alter von 65 Jahren eine Demenz [2]. Dennoch spiegelt sich dieses Wissen über das stark erhöhte Demenzrisiko und den daraus resultierenden Bedarf bislang nicht in den Versorgungsstrukturen im deutschen Gesundheitssystem wider, obwohl in der UN-Behindertenrechtskonvention, die in Deutschland seit 2009 in Kraft ist, ein freier Zugang von Menschen mit einer Behinderung zu medizinischer Behandlung und zu Forschung garantiert wird [3] und diverse internationale Empfehlungen zur umfassenden Berücksichtigung von Menschen mit intellektuellen Beeinträchtigungen bzw. MmDS im Speziellen in Demenzstrategien vorliegen (u. a. [4, 5]). Trotzdem werden MmDS in der aktuellen deutschen S3-Leitlinie „Demenzen“ nicht erwähnt [6]. Arzneimittelstudien schließen MmDS in der Regel explizit aus [7]. Dies hat für den besonderen Personenkreis der MmDS zur Folge, dass Behandlungsempfehlungen für sie im deutschen Gesundheitssystem derzeit nur von den Erfahrungen in der Allgemeinbevölkerung abgeleitet werden können. Dabei sind die Diagnostik und Therapie der Alzheimer-Demenz bei MmDS mit Herausforderungen verbunden, die sich unterschiedlich im Vergleich zur Versorgung der Allgemeinbevölkerung bei Demenz gestalten [8]. So weisen MmDS beispielsweise ein erhöhtes Risiko auf für diverse Komorbiditäten, die als Ätiologie einer erworbenen kognitiven Störung infrage kommen [9]. Die Diagnosestellung ist zudem aufgrund des stark variierenden kognitiven Ausgangsniveaus der Betroffen erschwert und bedarf daher anderer Assessmentstrategien und auch anderer Screeninginstrumente als in der konventionellen Demenzdiagnostik [10].

Vor diesem Hintergrund befasst sich die Studie „(Zugang zu) Diagnostik und Therapie demenzieller Erkrankungen bei Menschen mit einem Down-Syndrom“ (DS-Demenz, 01VSF21030) mit der empirischen Untersuchung der Versorgungssituation von MmDS und demenziellen Erkrankungen in Deutschland und der Ableitung von gesundheitspolitischen Handlungsempfehlungen zur Verbesserung der medizinischen Versorgung dieser. Gegenstand dieses Artikels sind die Ergebnisse leitfadengestützter Expert:inneninterviews zur Erhebung von Versorgungsdefiziten in der Diagnostik und Therapie und möglichen Lösungsansätzen.

## Methodik

Zwischen Mai und August 2023 wurden insgesamt 14 Interviews mit Vertreter:innen drei verschiedener Stakeholder-Gruppen durchgeführt (Tab. 1).

**Tab. 1:** Interviewpartner:innen

Stakeholder-Gruppe	Anzahl	Erläuterungen
Patient:innen- und Selbsthilfevertretungen	4	2 Organisationen der Patient:innenvertretung 2 Angehörige von MmDS und Demenz mit Anbindung an Selbsthilfegruppen
Ärzt:innen	5	2 Hausärzte 3 Fachärzt:innen (Neurologie, Psychiatrie), davon 2 ärztliche Vertreter:innen aus Behandlungszentren für Erwachsene mit Behinderung (MZEB)
Wohlfahrtverbände als Träger von Wohn- und Arbeitsstätten	5	4 Vertreter:innen von Wohneinrichtungen eine Vertreterin einer Arbeitsstätte

Grundlage der Interviews waren an die Stakeholder-Gruppe angepasste und auf Basis eines Scoping Reviews und einer Routinedatenanalyse erstellte teilstrukturierte Gesprächsleitfäden. Bei der Konstruktion des Leitfadens wurde nach der SPSS-Methode nach Helfferich vorgegangen [11]. Tab. 2 stellt exemplarisch und stichpunktartig den Leitfaden für Hausärzt:innen dar. Die hier dargestellten Ergebnisse fokussieren ausschließlich auf die Bereiche Diagnostik und Therapie. Aspekte des Zugangs sowie Schnittstellen in der Patient Journey sind nicht Gegenstand dieses Artikels, sondern werden separat publiziert.

**Tab. 2:** Leitfaden für Hausärzt:innen (verkürzt)

Themenblock	Inhalte der Fragen (Fokus immer MmDS)
A) Einleitungsfrage	1. Besonderheiten bei MmDS als Thema in der hausärztlichen Versorgung
B) (Zugang zur) **Diagnostik**	2. Typischer Diagnostikprozess, Unterschiede zu Menschen ohne DS
	3. Hürden und Hemmnisse
	4. Einschätzung zu notwendigen Änderungen
C) (Zugang zur) **Therapie**	5. Therapiemaßnahmen, Unterschiede zu Menschen ohne DS
	6. Einsatz von Arzneimitteln
	7. Einsatz von Heilmitteln
	8. Einschätzung zur Angemessenheit der Versorgung
	9. Hürden und Hemmnisse
	10. Lösungsansätze zur Beseitigung von Versorgungsdefiziten

Die Interviews wurden von einem Moderatorinnenteam per Videokonferenz über MS Teams (Microsoft) durchgeführt (Dauer: 30–60 min), audiovisuell aufgezeichnet sowie wörtlich transkribiert und pseudonymisiert [12]. Die Analyse erfolgte nach der inhaltlich-strukturierenden Variante der qualitativen Inhaltsanalyse in Anlehnung an Mayring [13] und Weiterentwicklungen durch Kuckartz [14]. Die Kodierung mittels MAXQDA wurde nach dem Vieraugenprinzip qualitätsgesichert. Bei Meinungsverschiedenheiten erfolgte die Hinzunahme eines dritten Senior Researcher.

## Ergebnisse

### Versorgungsdefizite und Hürden in der medizinischen Versorgung

**Abb. 1:**
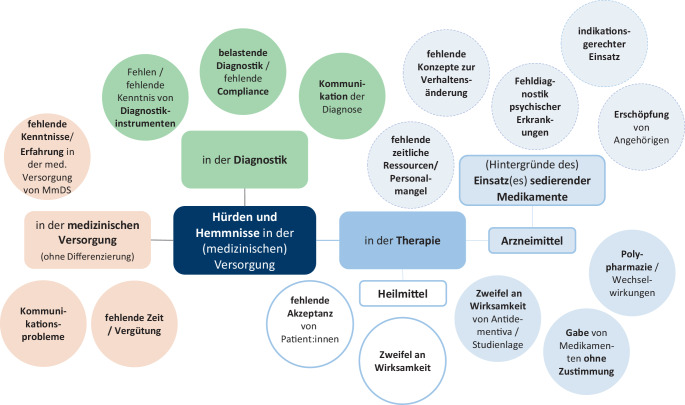
Versorgungsdefizite in der medizinischen Versorgung von MmDS und Demenz. (Quelle: eigene Darstellung)

Abb. 1 zeigt die in den Interviews genannten Versorgungsdefizite differenziert in die Bereiche Diagnostik und Therapie sowie in einen allgemeinen Bereich zur medizinischen Versorgung. Dieser umfasst Aspekte, die die Bereiche Diagnostik und Therapie gleichermaßen betreffen können.

#### Medizinische Versorgung

In jedem zweiten Interview wurden fehlende Erfahrungen bzw. fehlende Kenntnisse von Ärzt:innen in der Versorgung von MmDS genannt, da die meisten Ärzt:innen keine oder nur wenige Patient:innen mit DS regelmäßig behandeln und der Umgang mit dieser besonders vulnerablen Gruppe zudem nicht systematisch erlernt wurde. Ein MZEB-Vertreter sagte: „*Die Mediziner:innen, die sind da auch nicht … weitergebildet in dem Bereich Medizin für Menschen mit Behinderungen. Das ist ja auch echt ein Nischenfach und das wird ja auch nicht gelehrt im Studium*.“

Dies könnte auch eine Ursache für die mehrheitlich von Patient:innen- und Selbsthilfevertretungen sowie vereinzelt auch von Ärzt:innen selbst genannten Kommunikationsprobleme Letzterer sein (u. a. fehlende Informationsbereitstellung, fehlende Kommunikationskompetenz) (6/14).

Außerdem wurde das Fehlen zeitlicher Ressourcen bzw. das damit eng verknüpfte Fehlen einer auskömmlichen Vergütung angeführt (5/14). Insbesondere hausärztlich tätigen Ärzt:innen fehle die Zeit, sich neben dem Praxisalltag in Leitlinien zu verschiedenen Krankheitsbildern für unterschiedliche Zielgruppen einzulesen. Die Versorgung von Menschen mit einer Beeinträchtigung wurde als deutlich zeitaufwendiger beschrieben (z. B. bei der Durchführung bildgebender Verfahren zur Diagnostik einer Alzheimer-Demenz). Ein Hausarzt erläuterte: „*Dafür ist keine Zeit, wenn der … nicht stillhält …, das ist alles nichts, was die mögen … in ihren strukturierten Abläufen.*“ Ein vertragsärztlich tätiger Neurologe greift diesen Aspekt in einem größeren Zusammenhang auf: „*Das ist sehr zeitintensiv. Wir müssen sehr genau zuhören … und vor allem auch mit Betreuenden … sprechen. Das ist eine Form der Zuwendungsmedizin, die in unserem System so ein bisschen hinten runterfällt.*“ Auch die Refinanzierung der Gründung eines MZEB sowie die Vergütung der laufenden Patient:innenversorgung wird als herausfordernd bzw. defizitär beschrieben: „*Ein MZEB bringt keinen Profit. (…) für mich wäre Plus-Minus-Null total in Ordnung. (…) Wenn man ein MZEB aufbaut, muss man erstens die Räume vorweisen, zweitens die Geräte alle schon kaufen und drittens die Verträge der Mitarbeitenden vorlegen. Das kann kaum einer.“*

#### Diagnostik

Für den Bereich der Diagnostik wurde das Fehlen von bzw. die fehlende Kenntnis zu speziell auf die Zielgruppe zugeschnittenen neuropsychologischen Tests bei Ärzt:innen der Grund- und Regelversorgung genannt (7/14). Hervorgehoben wird die spezifische Herausforderung der Erhebung des individuellen kognitiven Ausgangszustandes. Ein vertragsärztlich tätiger Neurologe erläutert: „*Wir haben ja sonst … eine Vergleichspopulation und … Normwerte, und ein MmDS würde da nicht reinfallen. Das heißt, es gibt jetzt meines Erachtens keine speziellen Tests, die für Patienten mit DS validiert sind. Das dürfte auch schwerfallen, so etwas zu machen, weil ja natürlich die Varianz auch sowieso schon extrem ist*.“

Außerdem wurden Erwägungen über die Zumutbarkeit diagnostischer Maßnahmen wie z. B. einer Liquordiagnostik für die Patient:innen angeführt (5/14). Ein Hausarzt erläutert: „*Bei Untersuchungen, die dann natürlich auch für den Patienten … sehr anstrengend sind, weil sie zum Teil … sediert werden müssen, da überlegen wir uns natürlich schon zweimal, ob man das machen muss oder nicht.*“

Zudem wurde die Herausforderung der Kommunikation der Diagnose Demenz angeführt (1/14). Außerdem wurde berichtet, dass Angehörige von MmDS diagnostischen Maßnahmen z. T. sehr kritisch gegenüberstünden, was den diagnostischen Prozess weiter erschweren könne (1/14).

#### Therapie

Die Hälfte der Expert:innen nannte Zweifel an der Wirksamkeit von Antidementiva, da die Studienlage speziell für die Zielgruppe nur unzureichend sei oder in der jeweils eigenen Erfahrung der praktische Einsatz von Antidementiva eine nur geringe oder ausbleibende Wirkung erzielt habe. Ein vertragsärztlich tätiger Neurologe dazu: „*Wir machen das, aber ich kenne auch Kollegen, die glauben nicht daran. Die machen das auch gar nicht, weil sie nicht überzeugt sind.*“

Außerdem wurde kritisiert, dass Medikationspläne nicht hinterfragt oder hinsichtlich Wechselwirkungen überprüft würden (3/14). Eine MZEB-Vertreterin erläutert: „*Menschen mit Trisomie 21, wie auch sonst Menschen mit Intelligenzminderung, haben leider viel zu viele Medikamente. Ab fünf Medikamenten hebt jede Nebenwirkung die Wirkung gewissermaßen auf.*“

Sehr ausführlich wurde die Problematik des Einsatzes sedierender Medikamente bei MmDS und Demenz diskutiert. In fast allen Interviews wurden als Hauptgründe dafür ein Personalmangel und die damit einhergehenden fehlenden zeitlichen Ressourcen der Mitarbeitenden in Pflege- bzw. Wohneinrichtungen genannt. Eine Vertreterin aus dem Wohnbereich berichtet: „*Ich könnte mir vorstellen, dass es tatsächlich in vielen Fällen einfach so ist, dass man dadurch versucht, die Menschen so ein bisschen ruhiger zu stellen, anstatt, ja andere Maßnahmen zu suchen, die vielleicht zeitaufwendiger sind.*“

Damit einher geht auch das Fehlen individueller Konzepte zur Verhaltensänderung, welches als Grund für den Einsatz solcher Medikamente genannt wurde (5/14).

Ein dritter Grund war die Fehldiagnostik von Demenzsymptomen als Symptome psychischer Erkrankungen (5/14). Eine MZEB-Vertreterin erläutert: „*Es gibt ja so einen Wahlspruch, Menschen mit Intelligenzminderung haben ca. 60–80* *% mehr oder häufiger psychiatrische Erkrankungen. Daran halten alle fest, auch gute Psychiater (…) An Demenz wird wenig gedacht, viel zu wenig.*“

Im Fokus für den familiären Bereich stand die z. T. sehr hohe Belastungssituation für pflegende Angehörige. Eine Vertreterin der Patient:innenperspektive dazu: „*Angehörige sind teilweise einfach in Not, weil sie überhaupt nicht mehr zur Ruhe kommen, nachts nicht schlafen können und manchmal auch aggressives Verhalten da ist.*“

Wenngleich die Häufigkeit des Einsatzes sedierender Medikamente bei MmDS und Demenz auffällig hoch ist, kann es jedoch auch und gerade in dieser Population Situationen geben, die den Einsatz derartiger Medikamente notwendig machen (5/14). Ein vertragsärztlich tätiger Neurologe erläutert dazu: „*Ein Antipsychotikum ist dann möglicherweise indiziert, wenn ein Patient sehr schwer verwirrt ist oder verhaltensauffällig und das eine große Belastung ist. Benzodiazepin in der Regel dann auch in der Institution, wenn Sie das nicht anders deeskalieren können. … Wie gesagt, sollte man das in einer Notsituation machen und nicht chronisch, weil wir dann die Abhängigkeitsproblematik haben. … Aber die Rechtfertigung geht nie über dieses Thema Demenz.*“

Zu hohe Anforderungen der Therapie an die Patient:innen könnten zu einer Verweigerungshaltung dieser führen (1/14). Ein vertragsärztlich tätiger Neurologe äußerte Zweifel an der Wirksamkeit von Heilmitteln: „*Die Idee, Sie gehen zum Arzt, und der verordnet dann mal 30* *min Ergotherapie, was soll das denn bringen? … Das ist sehr schwierig.*“

**Abb. 2:**
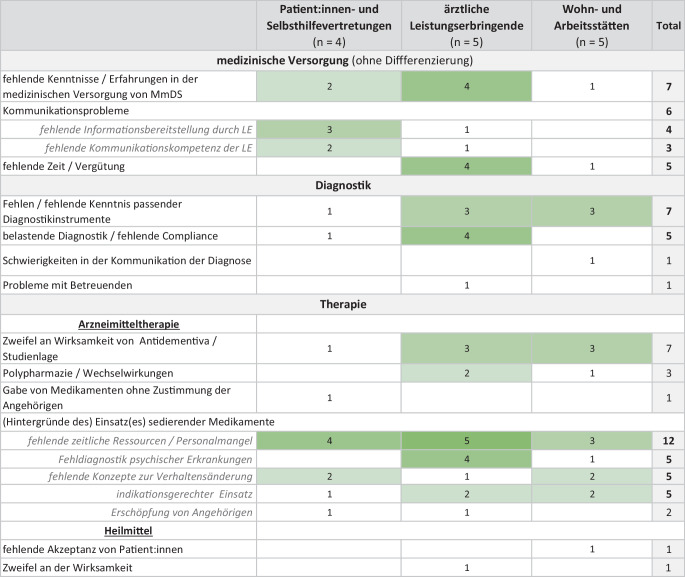
Versorgungsdefizite, differenziert nach Stakeholder-Gruppe, Intensität der grünen Hervorhebungen betont Häufigkeit der Nennungen

Patient:innen- und Selbsthilfevertretungen führten insbesondere Kommunikationsprobleme und eine fehlende Erfahrung von Ärzt:innen in der medizinischen Versorgung von MmDS an (Abb. 2). Als Hauptgrund für den vermehrten Einsatz sedierender Medikamente stuften sie fehlende zeitliche Ressourcen ein.

Auch Ärzt:innen selbst sahen eine zentrale Herausforderung in den fehlenden Kenntnissen/der fehlenden Erfahrung von Kolleg:innen. Sie betonten zudem die fehlenden Ressourcen für eine adäquate Versorgung der Zielgruppe und führten als einen weiteren zentralen Grund für den vermehrten Einsatz sedierender Medikamente eine mögliche Fehldiagnostik psychischer Erkrankungen an.

Bei den Hintergründen des Einsatzes sedierender Medikamente wiesen Vertreter:innen von Wohn- und Arbeitsstätten auf die oft fehlenden Konzepte zur nichtpharmakologischen Verhaltensänderung als Alternative zu Medikamenten hin, berichteten jedoch gleichzeitig von Situationen, die aus ihrer Sicht Sedativa notwendig machen könnten.

### Verbesserungsvorschläge und Best Practice

#### Medizinische Versorgung

**Abb. 3:**
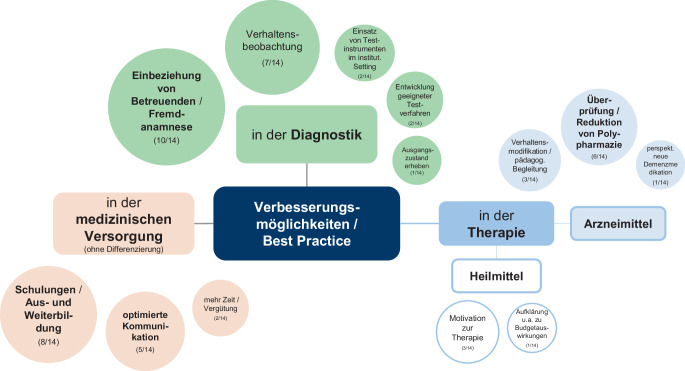
Verbesserungsmöglichkeiten und Best-Practice-Ansätze in der medizinischen Versorgung von MmDS und Demenz. (*Quelle*: eigene Darstellung)

Eine zentrale Forderung betrifft die Berücksichtigung der besonderen Bedürfnisse und Herausforderungen in der medizinischen Versorgung von MmDS und Demenz in der Aus‑, Fort- und Weiterbildung der am Versorgungsprozess Beteiligten (8/14; Abb. 3). Insbesondere Ärzt:innen sollten außerdem stärker über bereits in der Regelversorgung bestehende Möglichkeiten, beispielsweise durch die Zusammenarbeit mit MZEB, aufgeklärt werden.

Außerdem wurde die Notwendigkeit einer *angepassten Kommunikation bzw. eines angepassten Umgangs* mit Patient:innen bzw. Betreuenden betont (5/14). Eine MZEB-Vertreterin berichtet: „*Wir (…) schauen, dass die Atmosphäre gut ist. (…) Unsere Türen sind mit unterstützter Kommunikation (…) keine weißen Kittel (…) und einen guten Umgangston und nicht schwierige Worte, sondern eine Kommunikationsebene, die jeder verstehen kann.*“

Für den hausärztlichen Bereich wurde eine Anpassung der Vergütung für die Behandlung von Menschen mit intellektuellen Beeinträchtigungen gefordert: „*Im KV-System muss das auch irgendwie in der Vergütung sich niederschlagen, weil man kann nicht immer nur sagen, der Arzt ist Altruist.*“

#### Diagnostik

Betreuende sowie Mitarbeitende aus dem Arbeitsumfeld, die die Patient:innen schon länger kennen, sollten im Rahmen einer Fremdanamnese einbezogen werden. Ein vertragsärztlich tätiger Neurologe berichtet: „*Wir sind hier sehr stark … angewiesen, auf eine ganz saubere Anamnese und vor allem auch Fremdanamnese. (…) Die Betreuer kennen die und merken einfach, die kriegen viele Sachen nicht mehr hin, und die sind oft sehr gut ausgebildet, die Betreuer, und die können das sehr genau berichten.*“

Insbesondere Vertreter:innen aus dem Wohn- und Arbeitsbereich sowie die Patient:innen- und Selbsthilfevertretungen berichten zudem von der hohen Relevanz der aufmerksamen Verhaltensbeobachtung, die in vielen Einrichtungen praktiziert und dokumentiert wird, um Symptome bzw. veränderte Verhaltensweisen frühzeitig zu erkennen (7/14). In diesem Zusammenhang wurde vorgeschlagen, Testinstrumente oder vereinfachte Fragebögen regelmäßig durch formell Pflegende im institutionellen Setting einzusetzen (2/14).

Ein Hausarzt sowie eine Vertretung aus dem Bereich der Wohnbereiche wünschten sich ein „Tool“ zur Diagnoseunterstützung. Zudem wurde beschrieben, dass sich insbesondere die hausärztliche Versorgung durch die oft langjährige Betreuung für die Erhebung eines Ausgangszustandes bei MmDS besonders gut eignen könnte.

#### Therapie

Für die Therapie wurde die Bedeutung der regelmäßigen Überprüfung und Reduktion von Multimedikation insbesondere von Ärzt:innen hervorgehoben. Zudem müssen Medikamente mit anticholinerger Wirkung vermieden werden. Ein hausärztlich tätiger Arzt berichtet: „*Ich denke, die ganze Therapie muss immer differenziert auf den Patienten gucken, ob der damit offensichtlich besser klarkommt. (…) Und das fragen wir uns alle viel zu wenig.*“

Außerdem wurde von allen Stakeholder-Gruppen der Einsatz alternativer Maßnahmen zur Verhaltensmodifikation empfohlen. Eine Vertreterin aus dem Wohnbereich erläutert: „*Dann ist das schon unsere pädagogische Aufgabe oder unsere Aufgabe im sozialen Umgang mit den Menschen, Lösungen zu finden und nicht zu sagen, so, hey, der nervt, mach mal eine Pille rein.*“

Schließlich wurde von einer MZEB-Vertreterin auch auf erwartete Veränderungen durch spezifischere Demenzmedikamente hingewiesen: „*Dem neuen Medikament wäre ich tatsächlich gegenüber offen, weil das genau, was an Menschen mit Trisomie 21 als wissenschaftlich interessant herausgefunden wird, (…) mit diesem Medikament wahrscheinlich sogar gebremst wird.*“

Für den Heilmittelbereich wurde die Information von Ärzt:innen zu den Möglichkeiten einer Langzeitverordnung sowie zur extrabudgetären Verordnung von Heilmitteln für diese Zielgruppe genannt. Außerdem wurde darauf hingewiesen, die Bedeutung des Heilmitteleinsatzes hervorzuheben und Patient:innen durch Einfühlungsvermögen zur Therapie zu motivieren.

## Diskussion

In der medizinischen Versorgung der Alzheimer-Demenz bei MmDS zeigten sich diverse Herausforderungen und Versorgungsdefizite. Fehlende fachliche Kenntnisse, aber auch fehlende Kenntnisse über den angemessenen Umgang bei Ärzt:innen wurden angeführt und werden auch in der internationalen Literatur häufig beschrieben (u. a. [15]).

Für die hier angeführte erschwerte Diagnostik wurden u. a. fehlende Kenntnisse in der Regelversorgung über für die Zielpopulation adaptierte und validierte Diagnostikinstrumente sowie fehlende Ressourcen, die dem angemessenen Umgang mit dieser vulnerablen Patient:innengruppe zuwiderlaufen, als Gründe genannt. Diese Faktoren, die die Diagnostik bei der Zielgruppe erschweren, werden auch in der Literatur genannt [16].

Außerdem wurden in dieser Teilstudie Hintergründe für einen erhöhten Einsatz sedierender Medikamente diskutiert (u. a. fehlende zeitliche Ressourcen, Fehldiagnostik psychischer Erkrankungen), die selbst von nahezu allen Ärzt:innen angeführt wurden und Hinweise auf Versorgungsmängel geben. Wenngleich sedierende Medikamente im Rahmen demenzieller Erkrankungen z. B. zur Behandlung von Eigen- oder Fremdgefährdung indiziert sein können, stellt sich hier die Frage, ob die Indikationsstellung in manchen Fällen (z. B. zur „Ruhigstellung“) zu weit gefasst wird. Dieser potenzielle Fehleinsatz sedierender Medikamente wird auch in der Literatur beschrieben [17], wobei nicht die dahinterliegenden Gründe eruiert wurden. Hier bietet diese Teilstudie einen Erkenntnisgewinn, da nur durch das Verstehen von Motiven Verbesserungen eingeleitet werden können.

Die wenigen Nennungen zur Heilmittelversorgung könnten auf den in der derzeitigen therapeutischen Versorgung nur geringen Einsatz dieser hinweisen. Der mangelnde Einsatz nichtmedikamentöser Interventionen wird nicht nur für MmDS im Speziellen beobachtet, sondern scheint ein generelles Problem in der hausärztlichen Versorgung von Menschen mit Demenz zu sein [18].

Da die Lebenserwartung von MmDS erheblich gestiegen ist und weiter steigt und mit ihr das Risiko, eine Demenz zu entwickeln, wird es immer wichtiger, angemessene Versorgungsstrukturen aufzubauen. Eine präzise Diagnose ist zentral, um eine frühzeitige symptomatische und sozialmedizinische Therapieplanung einleiten zu können und eine gute Prognose (u. a. Fortschreiten der Krankheit verzögern, Lebensqualität erhalten) zu ermöglichen. Dabei sollten insbesondere die hier angeführten Lösungsansätze und Verbesserungsmöglichkeiten Berücksichtigung finden und in ein multidisziplinäres und sektorübergreifendes Gesamtkonzept in Form von politischen Handlungsempfehlungen überführt werden. Eine zentrale Forderung war die Berücksichtigung der besonderen Bedürfnisse und Herausforderungen in der medizinischen Versorgung von Menschen mit intellektuellen Beeinträchtigungen in der Aus‑, Weiter- sowie Fortbildung der am medizinischen Versorgungsprozess beteiligten Berufsgruppen. Ein von der Bundesärztekammer anerkanntes Fortbildungs-Curriculum zum Thema „Medizin für Menschen mit intellektueller Beeinträchtigung oder mehrfacher Behinderung“ thematisiert zwar die besonderen Anforderungen an die Kommunikation und Interaktion sowie das Krankheitsbild Demenz in dieser Zielgruppe, ein spezifischer Bezug zu MmDS kann dem Curriculum jedoch nicht entnommen werden. Es richtet sich zudem an Fachärzt:innen, die vermehrt mit Menschen mit einer intellektuellen Beeinträchtigungen zusammenarbeiten und nicht an Hausärzt:innen oder Fachärzt:innen, die nicht auf diesen Personenkreis spezialisiert sind.

Diese Ergebnisse fließen im Rahmen der DS-Demenz-Studie in die Entwicklung gesundheitspolitischer Handlungsempfehlungen für die optimierte medizinische Versorgung von MmDS und Demenz ein.

## Limitationen

Die direkte Einbindung von MmDS wurde im Studienkonsortium intensiv diskutiert. Für die interviewten MmDS hätte dies sehr wahrscheinlich eine Erstinformation über ein sehr hohes Erkrankungsrisiko bedeutet. Daraus potenziell resultierende psychologische Unterstützungsbedarfe zur Verarbeitung dieser Information wären über die Möglichkeiten der Studie hinausgegangen. Außerdem ist hier das „Recht auf Nichtwissen“ über ein zukünftiges Erkrankungsrisiko anzuführen. Um die Perspektiven der Betroffenen jedoch auch im Rahmen dieser Studie einzubinden, wurden parallel Interviews mit Betreuenden von MmDS in der Ambulanz für Alzheimer bei DS der Ludwig-Maximilians-Universität München durchgeführt, die diese im konkreten Zusammenhang mit einer vermuteten oder diagnostizierten demenziellen Erkrankung aufsuchten. Diese Ergebnisse werden separat publiziert.

Die hier gewählte qualitativ und beschreibend angelegte Methodik ermöglicht keine quantitativen Aussagen über die den Stakeholder-Gruppen zugeordneten Erkenntnissen. Der in dieser Teilstudie gewählte Ansatz, ergänzend die Anzahl der Interviews zu nennen, soll daher nur als möglicher Hinweis zur Relevanz und nicht als quantitative Schlussfolgerung verstanden werden.

## Fazit für die Praxis

- Für die Gabe nichtindizierter sedierender Medikamente mit dem Ziel der Ruhigstellung bei MmDS gab es Hinweise; dies würde gegen rechtliche und ethische Vorschriften verstoßen.
- In der medizinischen Versorgung von Menschen mit intellektuellen Beeinträchtigungen ist eine zuwendungsvollere und zeitintensivere Arbeitsweise essenziell.
- In der Aus‑, Weiter- sowie Fortbildung der beteiligten Berufsgruppen sollten die Bedürfnisse und Herausforderungen in der Versorgung von MmDS und Demenz berücksichtigt werden.

## Data Availability

Zu diesem Beitrag liegen keine Daten vor.
